# Effectiveness and Safety of a Prolonged Hemodynamic Support by the IVAC2L System in Healthy and Cardiogenic Shock Pigs

**DOI:** 10.3389/fcvm.2022.809143

**Published:** 2022-02-08

**Authors:** Clément Delmas, Jean Porterie, Géraldine Jourdan, Frank Lezoualc'h, Romain Arnaud, Stéphanie Brun, Hugo Cavalerie, Grégoire Blanc, Bertrand Marcheix, Olivier Lairez, Patrick Verwaerde, Jeanne Mialet-Perez

**Affiliations:** ^1^Institute of Metabolic and Cardiovascular Diseases (I2MC), UMR1297, National Institute of Health and Medical Research (INSERM), University of Toulouse, Toulouse, France; ^2^Intensive Cardiac Care Unit, Department of Cardiology, Rangueil University Hospital, Toulouse, France; ^3^Department of Cardiovascular Surgery, Rangueil University Hospital, Toulouse, France; ^4^Critical and Intensive Care Unit, Stromalab UMR 5273 CNRS/UPS-EFS-ENVT-INSERM U1031, Toulouse School of Veterinary Medicine, Toulouse, France; ^5^Department of Anesthesia, Intensive Care and Perioperative Care Medicine, University Hospital, Toulouse, France; ^6^ENVA/UPEC/IMRB-Inserm U955, Ecole Nationale Vétérinaire d'Alfort, Maisons-Alfort, France

**Keywords:** mechanical circulatory support, IVAC2L, porcine model, safety, efficiency, cardiogenic shock

## Abstract

**Background:**

Mechanical circulatory supports are used in case of cardiogenic shock (CS) refractory to conventional therapy. Several devices can be employed, but are limited by their availability, benefit risk-ratio, and/or cost.

**Aims:**

To investigate the feasibility, safety, and effectiveness of a long-term support by a new available device (IVAC2L) in pigs.

**Methods:**

Experiments were carried out in male pigs, divided into healthy (*n* = 6) or ischemic CS (*n* = 4) groups for a median support time of 34 and 12 h, respectively. IVAC2L was implanted under fluoroscopic and TTE guidance under general anesthesia. CS was induced by surgical ligation of the left anterior descending artery. An ipsilateral lower limb reperfusion was created with the Solopath® system. Reperfusion was started after 1 h of support in healthy pigs and upon IVAC2L insertion in CS pigs. Hemodynamic and biological parameters were monitored before and during the whole period of support in each group.

**Results:**

Occurrence of an ipsilateral lower limb ischemia was systematic in healthy and CS pigs in a few minutes after IVAC2L implantation, and could be reversed by the arterial reperfusion, as demonstrated by distal transcutaneous pressure in oxygen (TcPO_2_) and lactate normalization. IVAC2L support decreased pulmonary capillary wedge pressure (PCWP) (15.3 ± 0.3 vs. 7.5 ± 0.9 mmHg, *p* < 0.001), increased systolic blood pressure (SBP) (70 ± 4.5 vs. 101.3 ± 3.1 mmHg, *p* < 0.01), and cardiac output (CO) (4.0 ± 0.3 vs. 5.2 ± 0.6 l/min, *p* < 0.05) in CS pigs; at CS onset and after 12 h of support, without effects on heart rate or pulmonary artery pressure (PAP). Non-sustained ventricular arrhythmias were frequent at implantation (50%). A non-significant hemolysis was observed under support in CS pigs. Bleedings were frequent at the insertion and/or operating sites (30%).

**Conclusion:**

Long-term support by IVAC2L is feasible and associated with a significant hemodynamic improvement in a porcine model. These preclinical data open the door for a study of IVAC2L in human ischemic CS, keeping in mind the need for systematic reperfusion of the lower limb and the associated risk of bleeding.

## Introduction

Cardiogenic shock (CS) is defined as an organ hypoperfusion secondary to the impaired cardiac output (CO). It comes in different forms, ranging from a mild hypoperfusion or low CO, to an array of deep shock with secondary multiorgan failure. Criteria for defining cardiogenic shock (CS) in humans associate with systolic blood pressure (SBP) <90 mmHg or need for vasopressors; pulmonary congestion or increased left ventricular pressures; and organ malperfusion signs (1). Myocardial infarction (MI) and its complications are the main causes of CS in ~40–60% of cases (1–3). It is a common medical situation affecting 60,000–70,000 patients per year in Europe (4) and 5–15% of acute coronary syndromes (2).

Despite therapeutic advances involving medication, early revascularization, and initial close supervision in specialized intensive care units (ICUs) of patients with MI, prognosis remains poor with immediate mortality of 40–50% of cases (2). Optimal management combines coronary reperfusion with classic intensive care management to restore tissue oxygenation through volume management, mechanical ventilation, vasopressors, and/or inotropes (1, 5, 6).

In 15–20% of the cases designated as refractory CS, classical drugs are ineffective. In these cases, mechanical circulatory supports (MCS) have been developed for a dual role: first, to ensure CO to prevent or treat organ failure secondary to hypoperfusion and second, to allow the unloading of the ventricles and to promote myocardial recovery (5, 7).

Regarding circulatory support, data are conflicting and large-scale randomized studies are lacking. After the IABP-SHOCK 2 study (8), the interest of intra-aortic balloon pump (IABP) has been severely questioned and its position in the management of CS was clearly downgraded in class IIIB for routine use (9).

Given the heterogeneity of clinical presentations, the diversity of therapeutic approaches in the absence of large-scale randomized studies with unequivocal results, the United States and European recommendations remain poorly directive and precise concerning the type and the timing of MCS (level of recommendation IIBC for European Society of Cardiology; and IIAb for American, Indian, and Canadian Heart Failure and Interventional Cardiology societies) (7, 9–12).

In this context, new support devices are emerging, and the difficulty for the clinician is to find assistance tailored to the patient whatever in terms of effectiveness (left ventricular unloading and output delivery) and safety (bleeding, vascular complications, hemolysis, infection, and reliability). Furthermore, in the current economic climate, their benefit-risk ratio must necessarily be weighted by the costs generated. Consequently, preclinical research is essential for the implementation of the necessary knowledge on the topic.

The IVAC2L (PulseCath® Amsterdam; The Netherlands) is a new MCS which has obtained the European conformity (CE) marking in the indication of high-risk percutaneous coronary intervention (PCI) in 2015 (13). Device is inserted into the left ventricle through retrograde crossing of the aortic valve from a percutaneous femoral approach and is connected to an extra-corporeal membrane pump. Using EKG triggering, blood is aspirated from the left ventricle through the inlet tip into the membrane pump during systole and ejected from the pump through the catheter valve into the ascending aorta during diastole, resulting in a pulsatile support, with additional output, systolic unloading of the left ventricle, and diastolic counter-pulsation.

At present, use of IVAC2L is limited to case reports and case series (14–16) for few minutes during high-risk PCI. It seems to generate a rate of up to 1.4 l/min and to decrease the pulmonary capillary wedge pressure (PCWP). Its price, lower than the other available devices, and its compatibility with any standard and widely available IABP drive-console which is widely available, potentially makes this device more accessible. However, until now, it was never studied for extended support periods beyond 2 h, nor in the CS indication. An initial personal *ex-vivo* evaluation of the IVAC2L circulatory support system and its insertion kit (Solopath®) was performed and highlighted a high risk of limb ischemia secondary to the 19 Fr arterial cannulation justifying associating an arterial reperfusion.

The present study was developed to investigate the feasibility, safety, and efficiency of a long-term support by this new available device IVAC2L (PulseCath®) in a porcine model of CS.

## Materials and Methods

All experiments were reviewed and approved by the national INSERM French Ethics Committee for animal experimentation (CE201609131807621V8). The procedure for the care and sacrifice of study animals was in accordance with the European Community Standards on the Care and Use of Laboratory Animals. Details on materials and methods are described in Supplementary Material.

### Circulatory Support

The iVAC 2L (PulseCath® Amsterdam; The Netherlands) has 3 essential components: (1) the extra-corporeal membrane pump containing a blood chamber and an air chamber separated by a thin flexible membrane, (2) a bi-directional flow catheter, and (3) a patented rotating two-way valve. The blood chamber is connected to the bi-directional flow catheter and the air chamber to a mainstream intra-aortic balloon pump (IABP) console. The total chamber volume is 40 ml and the pump can eject a maximum volume of blood of 21 mL. The bi-directional flow catheter has a total length of 105 cm and a 17 Fr (5.9 mm) outer diameter. At 6 cm from the inlet tip, the catheter has an integrated two-way valve that pivots around two axes.

### Animals

All animals investigated (6 healthy and 6 CS pigs) were adult male pigs (*Sus scrofa domestica*) of 6–8 months old (mean weight 80–100 kg).

After initial intramuscular pre-medication (ketamine and midazolam), anesthesia was induced and maintained by continuous intravenous association of midazolam—propofol—sufentanyl—cisatracurium besilate. During experiments, pigs were under invasive mechanical ventilation under assisted volume-controlled mode (tidal volume 8 ml/kg, positive end-expiratory pressure (PEEP) 5 cm H_2_O, initial FiO_2_ 100%, and then, adjusted to a saturation of 94–96%). Vascular filling and vasopressors were used as needed to maintain mean blood pressure (MBP) between 65 and 80 mmHg.

Antiarrhythmic preparation was made by amiodarone to prevent the expected risk of ventricular arrhythmias.

### IVAC2L Insertion and Management

The right common femoral artery (FA) was exposed through an oblique incision in the groin crease. Then, an anterograde puncture of the FA allowed the insertion of a 7 Fr Super Arrow-Flex^TM^ sheath (Teleflex, Wayne, PA, USA). Next, a retrograde puncture was performed above the previous site to insert the 19 Fr SoloPath^TM^ balloon expandable transfemoral system (Terumo Corp., Tokyo, Japan). Finally, Super Arrow-Flex^TM^ sheath was connected to the Solopath sheath to perfuse the right inferior limb.

The IVAC2L system implantation was performed as previously described through the aortic valve in the left ventricle under fluoroscopic and TTE guidance (13, 17). The extra-corporeal membrane pump was connected to the IABP console (Arrow ACAT 2 Wave) which was then activated and synchronized to the animal thanks to the EKG and invasive blood pressure monitoring.

In the case of ventricular arrhythmia, additional intravenous injections of antiarrhythmic were made (amiodarone +/− lidocaine bolus).

An initial intravenous bolus of unfractionated heparin (UFH) (30 UI/kg) was made at reperfusion insertion time and anticoagulation target (activated clotting time between 200 and 250 s) was then maintained by continuous intravenous infusion (2,500–6,000 UI/h).

### Cardiogenic Shock Induction

After median sternotomy and pericardiotomy, the left anterior descending (LAD) was isolated and a ligation site was located at the level of the second diagonal branch. Before ligation, the animals received a new bolus of amiodarone to prevent possible arrhythmias. Then, MI was induced by the ligation of the LAD (4-0 Prolene wire). After ligation, segment ST-elevation on electrocardiography and a color change in the ventricular myocardium occurred in all animals. Two pericardial drains were placed and incision was closed in anatomic layers. Cardiogenic shock was defined according to usual definition (1). If CS was not obtained in few minutes, an additional and proximal ligation of the LAD was made.

### Monitoring

A non-invasive monitoring of heart rate, blood oxygen saturation, and EKG was continuously performed. Invasive SBP, diastolic blood pressure (DBP), and MBP were monitored by using an arterial catheter. A Swan-Ganz catheter monitored pulmonary artery pressure (systolic pulmonary artery pressure [sPAP]), mean pulmonary artery pressure [mPAP], diastolic pulmonary artery pressure [dPAP], PCWP, and CO using the thermodilution approach (Vigilance II, Edwards®).

Lower limb perfusion was clinically assessed (heat, cutaneous coloration time) and a continuously transcutaneous pressure in oxygen (TcPO_2_) monitoring was also used (TCM 400-2, Radiometer SAS®, France) during experiments.

Respiratory (arterial and venous blood gases) and renal (creatinine, kaliemia, natremia, and chloremia) functions, systemic and distal limb perfusion (lactate), and hemostase (TQ, aPTT) were repeatedly monitored by a relocated biology monitor (EPOC, CoagPoc, EDGE®). Other biological samples were frozen to realized specific *post-hoc* analysis (LDH and troponin).

### Statistical Analyses

Statistical evaluation and figures were performed with Graph Pad Prism version 6.0 for Windows (Graph Pad Software, La Jolla, CA, USA). Normal distributed continuous variables are expressed as the means ± SEM. Categorical variables are expressed as absolute numbers and percentages.

**Figure 1:**
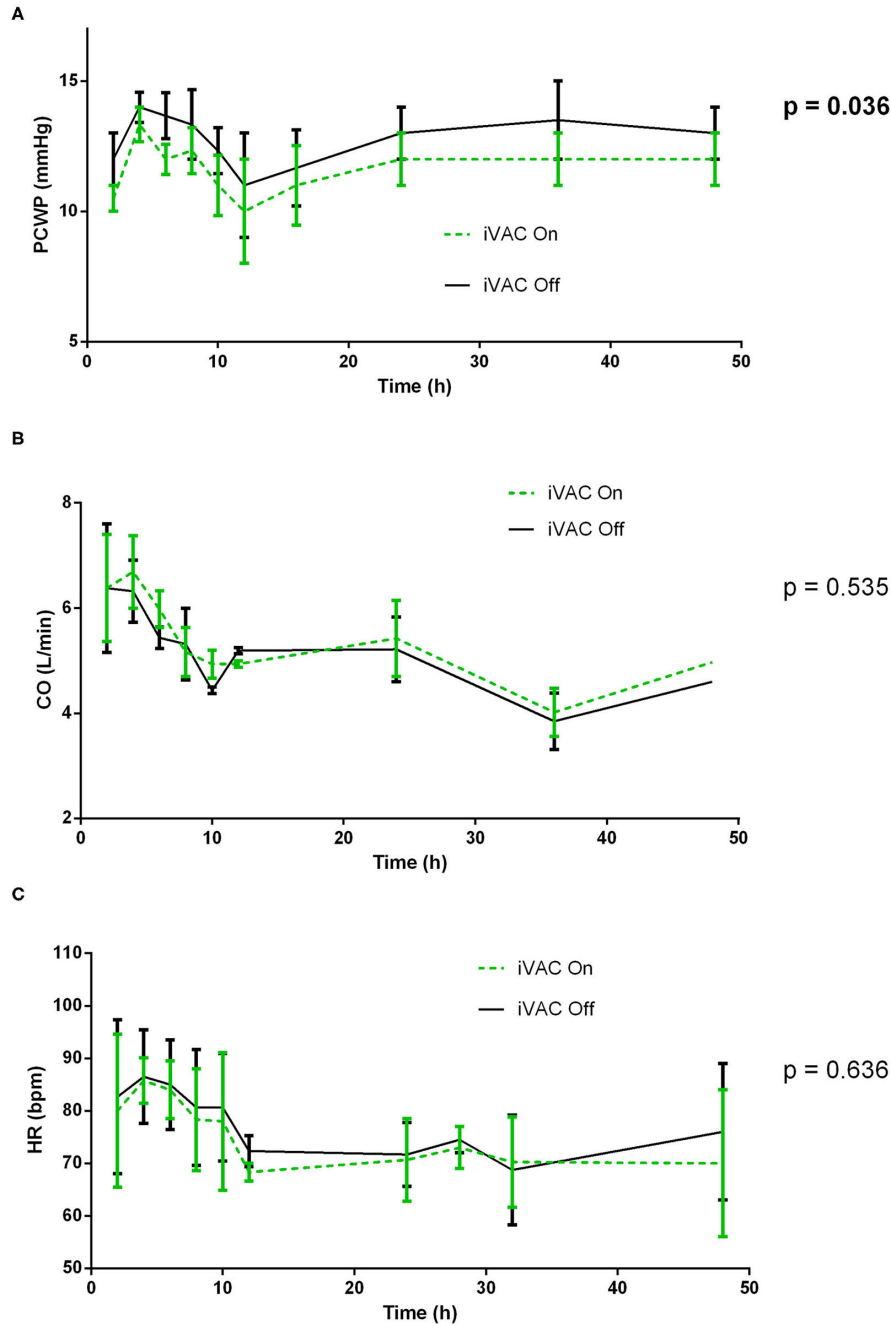
Comparative analysis of pulmonary wedge pressure **(A)**, cardiac output (CO) **(B)**, and heart rate **(C)** with and without IVAC2L support in healthy pigs. At different times (H0, H2, H4, H6, H8, H10, H12, H16, H24, H36, and H48), the IVAC2L support was deactivated by turning Off the extra-corporal pump during 5 min and then, reactivated until next time point. Invasive and non-invasive hemodynamic parameters were recorded throughout the procedure and until 48 h. Parameters are presented at each time points by mean ± SEM (*n* = 6). The values of *p* were obtained by comparing On and Off values for each parameter. CO, cardiac output; HR, heart rate; PCWP, pulmonary capillary wedge pressure; SEM, standard error of mean.

**Figure 2:**
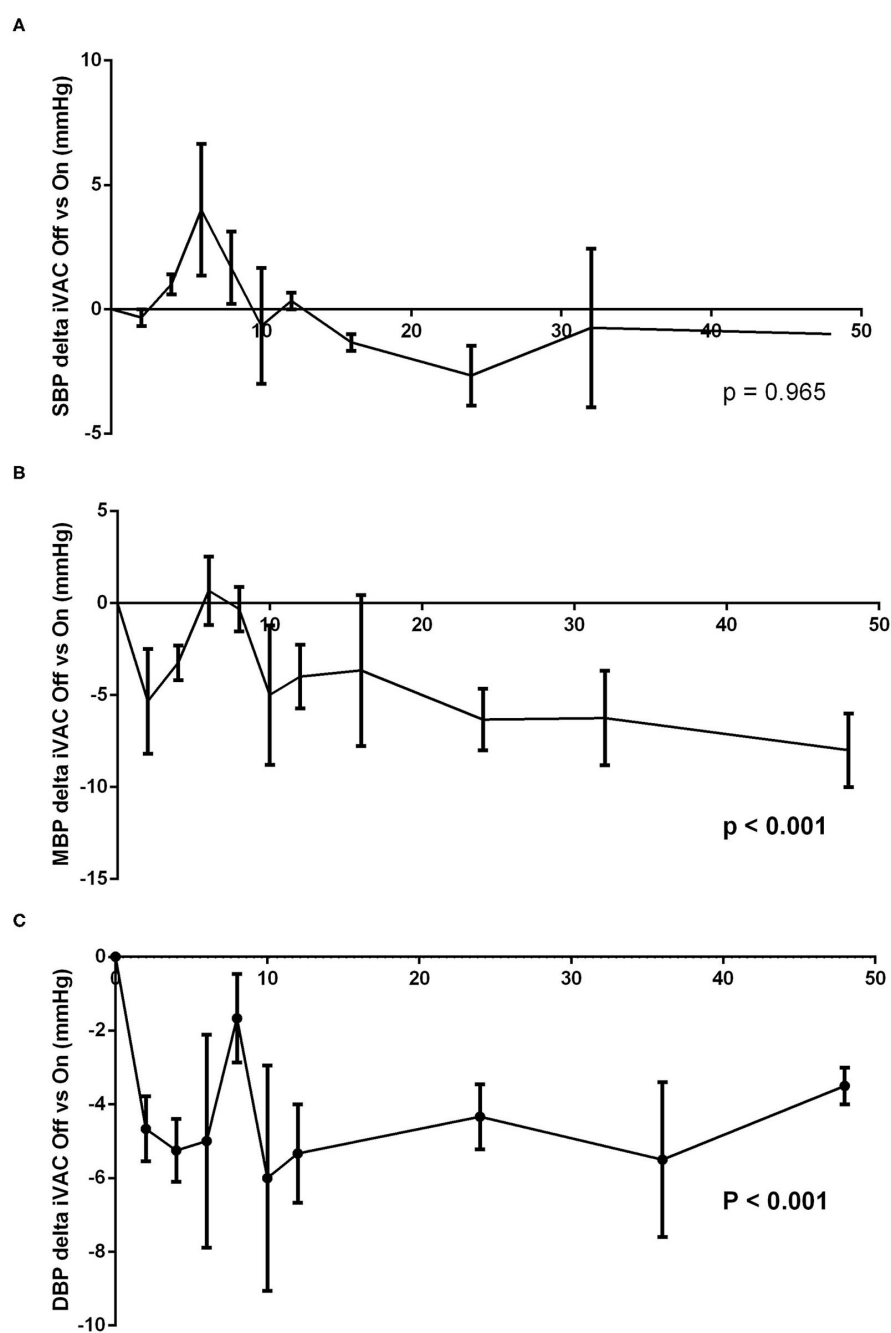
Analysis of difference between systolic blood pressure (SBP) **(A)**, mean blood pressure (MBP) **(B)**, and diastolic blood pressure (DBP) **(C)** with and without IVAC2L support in healthy pigs. At different times (H0, H2, H4, H6, H8, H10, H12, H16, H24, H36, and H48), the IVAC2L support was deactivated by turning Off the extra-corporal pump during 5 min and then, reactivated until next time point. Invasive and non-invasive hemodynamic parameters were recorded throughout the procedure and until 48 h. Data are presented at each time points by the delta pressure mean between IVAC2L On and Off ± SEM (*n* = 6). The values of *p* were obtained by comparing the delta pressure On and Off for each parameter. DBP, diastolic blood pressure; MBP, mean blood pressure; SBP, systolic blood pressure; SEM, standard error of mean.

**Figure 3:**
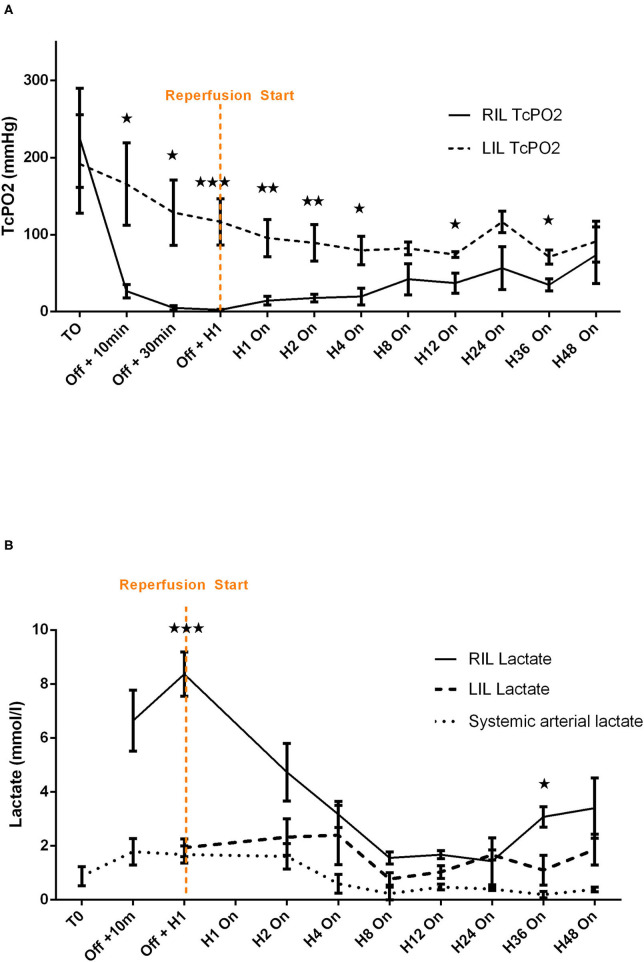
Comparative analysis of transcutaneous pressure in oxygen **(A)** and lactate **(B)** evolution before and after reperfusion start in healthy pigs. Transcutaneous pressure in oxygen (TcPO_2_) are presented at each time points by mean ± SEM (*n* = 6). The values of *p* were obtained by comparing RIL (IVAC2L) and LIL. ^⋆^*p* < 0.05; ^⋆⋆^*p* < 0.01, and ^⋆⋆⋆^*p* < 0.001. T0, basal measure after sedation; H1, 1 h post reperfusion On; H4, 4 h after reperfusion On; H8, 8 h after reperfusion On; H12, 12 h after reperfusion On; H24, 24 h after reperfusion On; H36, 36 h after reperfusion On; H48, 48 h after reperfusion On; LIL, left inferior leg; *n*, number of pigs; Off, Solopath inserted but reperfusion Off; RIL, right inferior leg; SEM, standard error of mean; TcPO_2_, transcutaneous pressure in oxygen.

In healthy pigs, comparisons between values obtained at different time points with On and Off support were made by a 2-way ANOVA (Figures 1–**3**). Moreover, hemodynamic parameters obtained during support On vs. Off, were averaged and compared with each other by an unpaired *t*-test (Supplementary Table 1).

**Figure 4:**
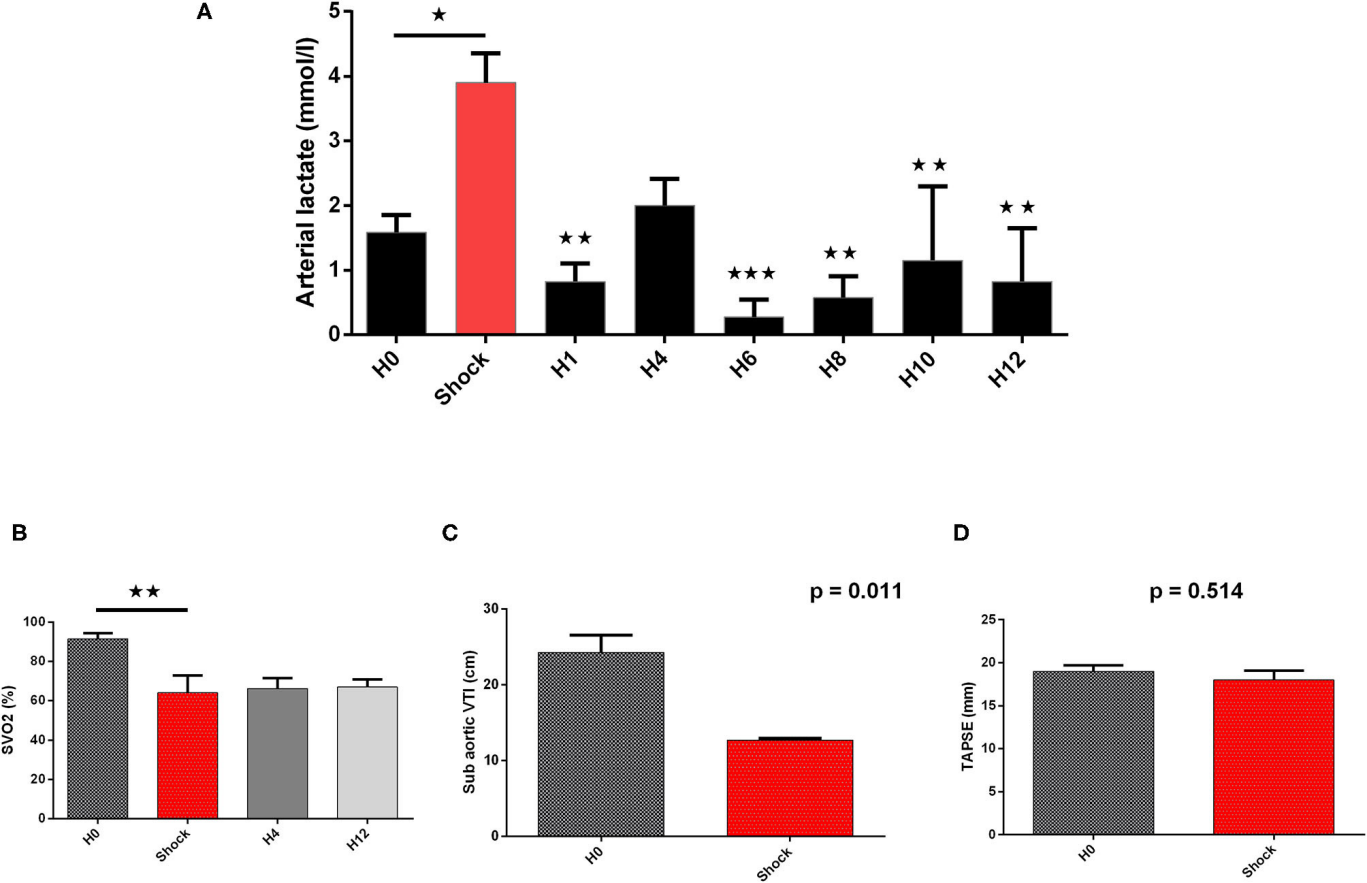
Biological and echographical markers of cardiogenic shock (CS) evolution. Lactate and SVO_2_ are presented at baseline (H0), at CS onset, and after IVAC2L implantation as mean ± SEM (*n* = 4). The values of *p* were obtained by comparing each point to CS point for arterial lactate and SVO_2_. Echographical parameters (TAPSE and sub-aortic VTI) were recorded at baseline and at CS onset before IVAC2L implantation and are reported as mean ± SEM. The values of *p* were obtained by comparing sub-aorticVTI and TAPSE. ^⋆^*p* < 0.05; ^⋆⋆^*p* < 0.01, and ^⋆⋆⋆^*p* < 0.001 vs. shock. H0, baseline; H1, 1 h post CS; H4, 4 h after CS; H12, 12 h after CS; SEM, standard error of mean; SVO_2_, venous oxygen saturation; TAPSE, tricuspid annular systolic excursion; VTI, velocity time integral.

**Figure 5:**
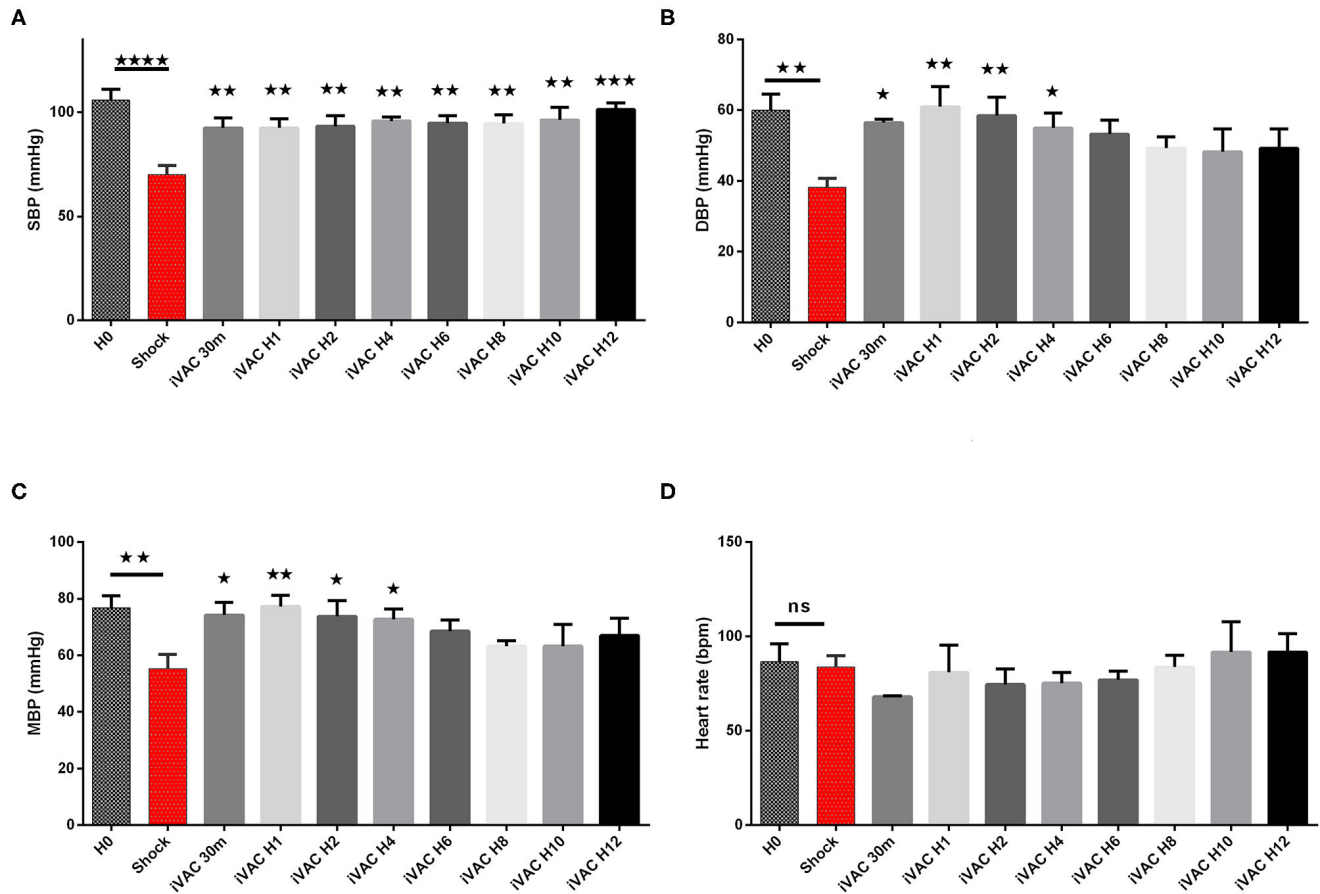
Hemodynamic parameters of IVAC2L support efficiency in CS pigs. Systolic blood pressure **(A)**, diastolic blood pressure **(B)**, mean blood pressure **(C)** and heart rate **(D)** are presented here. Parameters are presented as mean ± SEM (*n* = 4). The values of *p* were obtained by comparing the first baseline data to CS data, and second by comparing CS to others time points under IVAC2L support to CS onset (H4, H12). ^⋆^*p* < 0.05; ^⋆⋆^*p* < 0.01, ^⋆⋆⋆^*p* < 0.001 vs. shock, and ^⋆⋆⋆⋆^*p* < 0.0001. H0, baseline; H1, 1 h post CS; H4, 4 h after cardiogenic shock; H12, 12 h after CS. DBP, diastolic blood pressure; H0, baseline; HR, heart rate; MBP, mean blood pressure; SBP, systolic blood pressure; SEM, standard error of mean.

**Figure 6:**
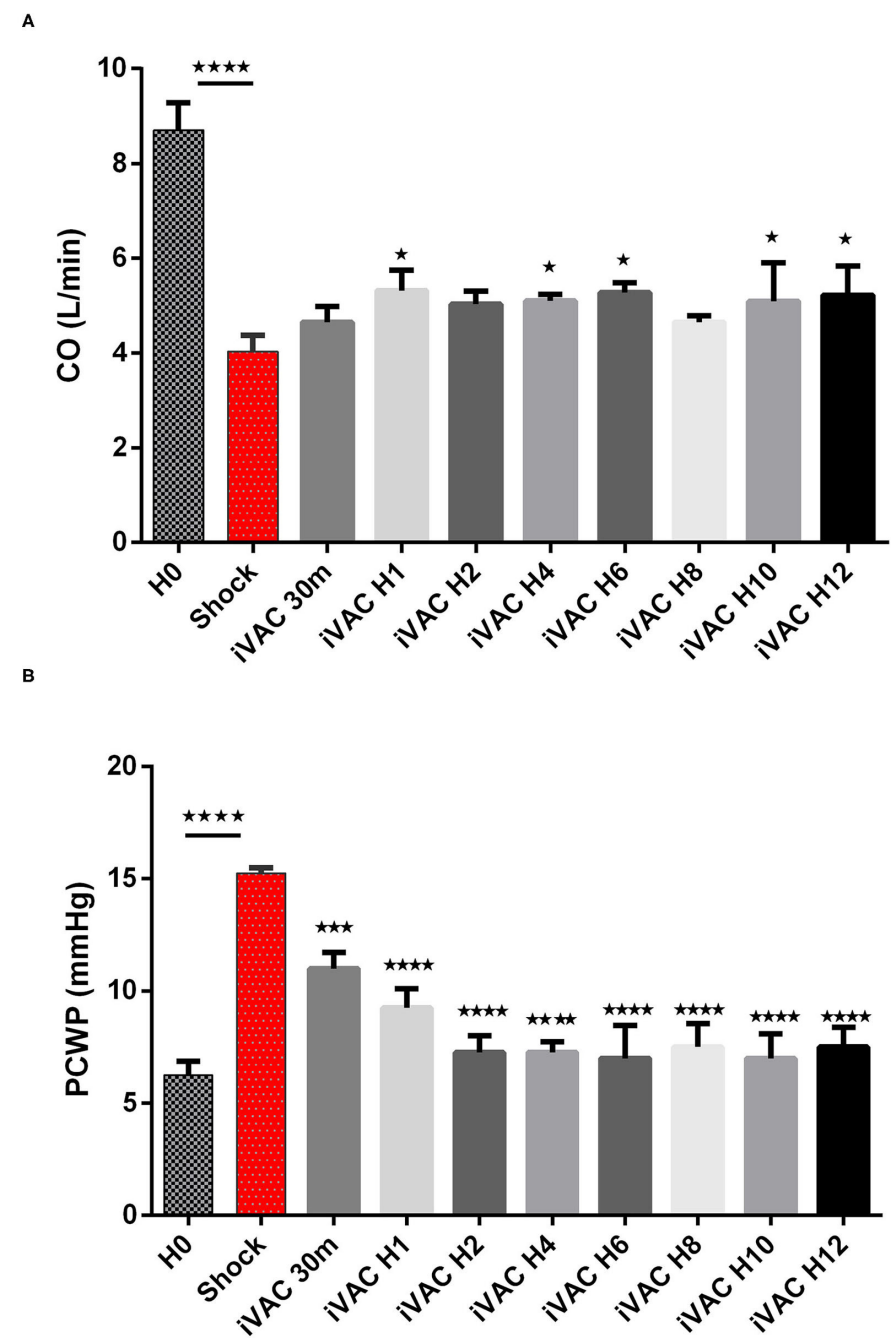
Evolution of cardiac output **(A)** and PCWP **(B)** in CS pigs before and after IVAC2L support. Parameters are presented as mean ± SEM (*n* = 4). The values of *p* were obtained by comparing the first baseline data to CS data, and second by comparing CS to others time points under IVAC2L support to CS onset (H4, H12). ^⋆^*p* < 0.05, ^⋆⋆⋆^*p* < 0.001 vs. shock, and ^⋆⋆⋆⋆^*p* < 0.0001. CO, cardiac output; H0, baseline; H1, 1 post CS; H4, 4 h after CS; H12, 12 h after CS; PCWP, pulmonary capillary wedge pressure; SEM, standard error.

In CS pigs, comparison between pigs at different time points was made by a paired 1-way ANOVA (Table 1, **Figures 4**–**6**). Moreover, main clinical, biological, and hemodynamic parameters were averaged at baseline, CS, and on support (H4 and H12). They were compared by using a 1-way ANOVA followed by Dunett's test for multiple comparisons. Comparisons of baseline vs. CS, H4 vs. CS, and H12 vs. CS were reported (Table 1).

**Table 1:** Comparative analysis of pigs' characteristics before and during cardiogenic shock (CS) under IVAC2L support.

	**Basal H0**		**Cardiogenic Shock**		**Under support H4**		**Under support H12**		***p*-value Basal vs CS**	***p*-value H4 vs CS**	***p*-value H12 vs CS**
	**Mean**	**SEM**	**Mean**	**SEM**	**Mean**	**SEM**	**Mean**	**SEM**			
**HEMODYNAMIC DATA**											
SBP (mmHg)	105.8	5.3	70.0	4.5	95.8	2.1	101.3	3.1	**0.001**	**0.01**	**0.003**
DBP (mmHg)	60.0	4.5	38.3	2.5	55.0	4.2	49.3	5.5	**0.017**	**0.042**	0.101
MBP (mmHg)	76.8	4.3	55.3	5.2	72.8	3.6	67.0	6.2	**0.01**	**0.026**	0.108
HR (bpm)	86.5	9.6	83.8	6.0	75.3	5.5	91.8	9.7	0.99	0.82	0.84
EtCO2 (mmHg)	39.5	4.9	31.0	2.9	32.5	3.6	30.5	2.4	**0.03**	0.9	0.99
**BIOLOGICAL DATA**											
Art pO2 (mmHg)	553.0	54.0	355.8	82.5	321.5	79.4	364.1	77.5	**0.02**	0.88	0.99
Art hemoglobin (dg/L)	8.2	0.6	7.6	0.6	7.3	0.8	5.7	0.6	0.756	0.937	0.066
Bicarbonates (mmol/L)	32.8	0.9	27.1	1.0	29.7	1.2	25.9	2.2	0.05	0.467	0.895
Sodium (mmol/L)	146.5	3.0	145.8	2.5	143.8	1.9	144.8	2.1	0.843	0.240	0.710
Potassium (mmol/L)	3.7	0.2	3.8	0.1	4.1	0.1	4.4	0.4	0.899	0.689	0.689
Arterial lactate (mmol/L)	1.6	0.3	3.9	0.5	2.0	0.4	0.8	0.8	**0.030**	**0.036**	**0.010**
Creatinine (μmol/L)	141.2	26.2	162.2	20.9	158.8	26.3	181.9	18.0	0.275	0.985	0.321
**SWAN-GANZ DATA**											
sPAP (mmHg)	24.8	0.5	27.3	1.5	24.8	2.8	23.8	1.5	0.561	0.561	0.321
dPAP (mmHg)	15.8	1.3	14.3	1.3	12.8	1.9	11.3	1.1	0.781	0.350	0.142
mPAP (mmHg)	19.0	1.4	19.3	1.8	17.8	2.0	16.8	0.9	0.999	0.823	0.533
CO (L/min)	8.7	0.6	4.0	0.3	5.1	0.1	5.2	0.6	**0.002**	**0.033**	0.173
PCWP (mmHg)	6.3	0.6	15.3	0.3	7.3	0.5	7.5	0.9	**<0.0001**	**<0.0001**	**<0.0001**

*One-way ANOVA followed by Dunnett's multiple comparison tests were used to compare each point to CS onset. Art, arterial; CO, cardiac output; DBP, diastolic blood pressure; dPAP, diastolic pulmonary artery pressure; EtCO2, expired carbon dioxide; HR, heart rate; MBP, mean blood pressure; mPAP, mean pulmonary artery pressure; SBP, systolic blood pressure; SEM, standard error of mean; PCWP, pulmonary capillary wedge pressure; sPAP, systolic pulmonary artery pressure. Values in bold represent significant values (p < 0.05)*.

The value of *p* < 0.05 was considered significant. Following signs are used to symbolize the value of *p*: *p < 0.05, **p < 0.01, and ***p < 0.001.

## Results

### Long-Term Support by IVAC2L in Healthy Pigs

Kinking of iliac artery and device length limited the correct placement of the device in 1 pig, which was excluded from the analysis. The entire procedure was performed in 6 pigs with a median support time of 34 h (3 pigs were under support during 48 h).

#### IVAC2L Associated Hemodynamic Effect in Healthy Pigs

At different times, the IVAC2L support was deactivated by turning Off the extra-corporal pump during 5 min and then reactivated until next time point. Invasive and non-invasive hemodynamic parameters were recorded throughout the procedure and until 48 h. Activation of the IVAC2L support was associated with a non-significant increase in DBP and MBP (Supplementary Figure 1) and a significant decrease in PCWP (Figure 1, Supplementary Table 1). Analysis of delta pressure between IVAC2L On vs. Off showed significant increase in MDB and DBP (Figures 2B,C) without significant changes in SBP (Figure 2A), CO, heart rate, or PAP (Supplementary Table 1, Figure 1, and Supplementary Figure 2).

#### IVAC2L Associated Limb Ischemia and Effectiveness of Distal Reperfusion in Healthy Pigs

Limb ischemia appeared systematically in a few minutes with clinical evidence (loss of distal pulse, paleness, coldness, and mottling of the limb) and was confirmed by a rapid and significant fall of ipsilateral TcPO2 (Figure 3A) and a marked rise of ipsilateral distal lactate (Figure 3B).

The implementation of retrograde reperfusion of the ipsilateral limb by a 7 Fr catheter connected to the Solopath® system was possible in all animals. Few minutes of reperfusion was clinically effective for all animals with a disappearance of previous signs. A trend to TcPO_2_ normalization vs. contralateral limb (left lower limb) appeared in a few hours and remained stable after H4 until 48 h of support (Figure 3A), even if significant difference between right and left limb persisted (Supplementary Table 2). Lactate normalized rapidly with levels comparable with the contralateral limb after 1 h and remained stable until 48 h of support except at H36 (Figure 3B and Supplementary Table 3).

#### IVAC2L Associated Complications in Healthy Pigs

The IVAC2L implantation was associated with non-sustained ventricular arrhythmias in 3 (50%) cases which were resolved after anti-arrhythmic drugs. A significant increase in LDH appeared after IVAC2L placement and remained until 48 h (Supplementary Figure 3). One episode of clinical hemolysis was noted and resolved after IVAC2L repositioning and vascular filling. Two transient cavitation phenomena were observed due to malposition of the inflow cannula resolving after replacement under TTE guidance.

### Mid-term Support by IVAC2L in CS Pigs

Severe MI was induced in 6 pigs as demonstrated by significant EKG modification and troponin release (Supplementary Figure 4), but 2 pigs presenting with refractory electrical storm were excluded. Finally, CS was obtained for 4 pigs which were included in the study with a total support time of 12 h.

Cardiogenic shock was confirmed by a significant decrease in blood pressure (SBP and MBP) associated with a fall of CO assessed by the non-invasive (sub-aortic velocity time integral) and invasive (CO by Swan-Ganz) measures (Table 1, Figures 4–**6**). Significant mixed venous oxygen saturation (SVO_2_) decrease and lactate increase defined tissue malperfusion to complete the classic definition of CS which was obtained for all pigs (Table 1 and Figure 4). Right ventricle function parameters (tricuspid annular systolic excursion [TAPSE] and S' tricuspid (baseline: 8.5 ± 2.9 vs. shock: 7.5 ± 2.9 cm/s, *p* = 0.09) were preserved (Table 1 and Figure 4) and left ventricular failure was confirmed by an increase in PCWP (**Figure 6**), signing a classic left mono-ventricular CS associated with MI.

#### IVAC2L Associated Hemodynamic Effect in CS Pigs

The IVAC2L start was associated with a significant increase in DBP, MBP, and SBP compared with CS (Table 1 and Figure 5). A significant and persistent decrease in PCWP associated with an increase in CO was observed without significant change in PAP or heart rate (Table 1 and Figure 6). During IVAC2L support, we observed a significant decrease and normalization of arterial lactate which was maintained at H12 indicating a correction of tissue malperfusion even if SVO_2_ did not normalize completely (Table 1 and Figure 4).

#### Effectiveness of Distal Reperfusion in CS Pigs

The implementation of retrograde reperfusion of the IVAC2L ipsilateral limb was possible in all pigs and was clinically effective for all animals. No difference in distal right inferior limb lactate between H0 and during support, neither between the right and left limb was found (Supplementary Figure 5).

#### IVAC2L Associated Complications in CS Pigs

At implantation, premature ventricular beats or non-sustained arrhythmias appeared in 2 (50%) cases which resolved after IVAC2L replacement and anti-arrhythmic drugs. Initial non-significant LDH elevation was observed at H1 and rapidly resolved (Supplementary Figure 6). Bleedings were frequent at the IVAC2L implantation femoral site (*n* = 2, 50%) and/or intra-thoracic due to pleural or pericardial effusion needing surgical revision (*n* = 3, 75%) explaining hemoglobin drop during support (Table 1 and Supplementary Figure 7).

One episode of IVAC2L external ventricle and reperfusion thrombosis has been observed during the prolonged heparin withdrawal at H10 due to severe intra-thoracic bleedings at the operative site. This episode was associated with hemodynamic instability and lower limb ischemia justifying a replacement of the circulatory support and surgical revision.

## Discussion

Using a double approach with a healthy and a CS pig model, we demonstrate the feasibility and effectiveness of a long-term support by IVAC2L. Major points were demonstrated: (a) the occurrence of an ipsilateral lower limb ischemia was systematic and could be prevented or reversed by an arterial reperfusion created from the Solopath® system; (b) the IVAC2L increased blood pressure (MBP and DBP) and decreased PCWP in healthy pigs; (c) the IVAC2L increased blood pressure (SBP, MBP, and DBP), decreased PCWP and increased CO in CS pigs; and (d) IVAC2L support was associated with the hemolysis and bleeding which will require special attention when using prolonged support in humans.

To date, no animal or human data support the long-term use of IVAC2L circulatory support. Although the device has obtained a CE approval for short-term support during high-risk PCI in 2015, published experience with IVAC2L application in humans is scarce and concern <50 patients in few expert centers for high-risk PCI (14, 15). Only one case report addresses its place in the left ventricle unloading under veno-arterial extracorporeal membrane oxygenation support (18) and no published data describe its use in CS case. Therefore, there is a need for preclinical data before long-term use in humans, especially in case of CS situation.

To our knowledge, we conducted the first study on long-term support with IVAC2L circulatory device and confirmed the feasibility of IVAC2L implantation under fluoroscopic and transthoracic echocardiography guidance. As expected, and previously described, IVAC2L insertion was associated with premature ventricular beats and non-sustained ventricular arrhythmias in 1 on 2 cases justifying the premedication with anti-arrhythmic drugs (amiodarone and/or lidocaine) even in humans. In 1 case, the implantation was not effective due to device length limitation, as previously described in the first published human cases. A total length of 105 cm could be limiting in a case of vascular tortuosities or in tall subject and may be anticipated by the preprocedural multi-slice CT as suggested by some authors (14).

As expected, we confirmed the occurrence of an early ipsilateral lower limb ischemia secondary to the implantation of the IVAC2L through a Solopath® system in all pigs. Surprisingly, this predictable complication was not previously described in the rare published data (13–15). Although arteries of pigs and humans share the same wall structure, they differ by their size and this could explain that ischemia appeared early within few minutes in all our pigs whatever their hemodynamic situations (healthy or CS pigs). In addition, our longer median support time (34 and 12 h in healthy and CS pigs, respectively) differed with previous shorter support in high-risk PCI (between 67 and 122 min) (14, 15). This early and severe lower limb ischemia justified a lower limb reperfusion creation. After hemodynamic *ex-vivo* tests (data not shown), we chose to create it through the Solopath® system and demonstrated here its feasibility and also its safety. Short- and long-term clinical but also biological monitoring proved its efficiency until 48 h in healthy pigs (lactate normalization and TcPO_2_ progressive increase) and 12 h in CS pigs (right inferior leg lactate stability under support) indicating its possible use for long-term support in humans.

As shown previously, the hemodynamic effects in the absence of hemodynamic instability (healthy pigs) were less pronounced than in CS situation, although IVAC2L improved the PCWP, DBP, and MBP significantly (14, 15). We could assume that left ventricular unloading and CO generated by the counterpulsation created by IVAC2L, may be more pronounced in case of increased left ventricular pressure and low CO as demonstrated by higher significant effect in CS pigs (SBP, MBP, DBP, and CO). Moreover, the drop in PCWP in healthy pigs without associated change in CO, seems to show that IVAC2L takes over from the heart to provide part of the total flow. Some authors found a delay between IVAC2L start and hemodynamic effect that we do not observe here, but they reported invasive measures during the first minutes of support while our first evaluation was at 30 min. We found a trend toward a decrease effect on hemodynamics (DBP, MDP, and SBP) with time in healthy and CS pigs without changes on CO or PCWP. This may be secondary to vasoplegia and/or hypovolemia associated with the systemic inflammatory response syndrome and/or bleedings secondary to the IVAC2L itself but also to our pig model especially in CS situation (large sternotomy with intrathoracic inflammation and bleedings). Interestingly the beneficial hemodynamic effects that we observed translated into biological effects (systemic lactate normalization and trend to SVO_2_ correction), reflecting a correction of the organ failure induced by the initial shock. Finally, we did not demonstrate any change in the PAP, confirming the place of IVAC2L for left mono-ventricular failure and its support.

Bleedings were frequent secondary to the large vascular access needed, but above all to our CS model necessitating a large surgical approach (sternotomy, pericardiectomy, and ligation of the coronary vessels). Some severe intra-thoracic bleedings needing surgical revision and vascular filling explain the drop in hemoglobin in the CS group. So, anticoagulation explained, at least partly, the hemorrhagic complications but as demonstrated by thrombotic complication (thrombosis of the external ventricle and/or tube of the IVAC2L), curative anticoagulation is mandatory during support. There was no damage to cardiac structures (aortic or mitral valve, left ventricle, and pericardium) as assessed by the macroscopic analysis of the heart after explantation at the end of the procedure. As anticipated, LDH increased under support, especially early after implantation and in healthy pigs. This difference between healthy and CS pigs, may be explained by a time correlation with possible progressive increase under support (partial micro-thrombosis of the system, inflammation, and cumulative effects) but also by a probable higher turbulence and flow conflicts between the pump and the native heart in healthy pigs.

### Study Limitations

A first limitation is the apparent small sample size of our study, but in the present study, each pig serves as its own witness in the healthy group, and significant differences were observed in terms of limb ischemia—reperfusion, but also in terms of hemodynamic. Our low number of CS pigs does not allow a definitive conclusion but generates hypotheses justifying the pursuit of research on the IVAC2L support. Second, the initial period of 48 h of support was not achieved for all healthy pigs, but it is the first description of prolonged general anesthesia in pigs (maximum 54 h in our study) and the first IVACL2L support >2 h to our knowledge (median support of 34 and 12 h in healthy and CS pigs, respectively). Third, contrary to the previous publication of MCS in CS situation, we did not use the pressure volume loop to define CS and approach hemodynamic effects of our MCS (19, 20), but we use TTE and Swan Ganz catheter monitoring as it is used in clinical practice. Fourth, we used surgical ligation of the LAD artery to create our ischemic CS model. Our invasive open chest model was associated with bleeding and a systemic inflammatory response syndrome that may explain the relative instability of our hemodynamic results over time. Recently, a model of total percutaneous ischemic CS has been described based on an intracoronary ethanol injection titration under the fluoroscopic guidance (20, 21). It is possible that this model initially made it possible to visualize a more stable hemodynamic effect over time, by limiting the hypovolemia and vasoplegia secondary to bleeding. Finally, we did not compare IVAC2L with others available device as IABP, Impella pump (Abiomed, MA, USA) or veno-arterial extracorporeal membrane oxygenation (22, 23) but only studied long-term support with IVAC2L in two clinical situations (stable and unstable hemodynamic). Future research addressing these comparisons will be needed.

## Conclusion

Long-term support by IVAC2L system is feasible and associated with significant blood pressure and CO increase, more pronounced in the case of hemodynamic instability in a large animal model. These large animal data open the door to the study of IVAC2L in ischemic CS in humans, keeping in mind the need for systematic reperfusion of the lower limb and the associated risk of bleeding.

## Data Availability Statement

The raw data supporting the conclusions of this article will be made available by the authors, without undue reservation.

## Ethics Statement

The animal study was reviewed and approved by National INSERM French Ethics Committee for animal experimentation (CE201609131807621V8).

## Author Contributions

CD, OL, FL, BM, and JM-P contributed to the conception and design of the study. CD, OL, and JM-P contributed to the interpretation of the results. CD, JP, GJ, RA, SB, HC, GB, BM, OL, and PV carried out the animal experiments. CD, GJ, HC, GB, PV, and JM-P contributed to sample preparation. CD, GJ, RA, SB, HC, GB, and PV collected the data. CD and RA organized the database. CD performed the statistical analysis and wrote the first draft of the manuscript. All authors provided critical feedback and helped shape the research, analysis, and manuscript. All authors read and approved the submitted version.

## Funding

This study was funded by the Terumo Europe NV (3001 Leuven, Belgium).

## Conflict of Interest

CD received a grant from the Terumo to perform experiments. The remaining authors declare that the research was conducted in the absence of any commercial or financial relationships that could be construed as a potential conflict of interest.

## Publisher's Note

All claims expressed in this article are solely those of the authors and do not necessarily represent those of their affiliated organizations, or those of the publisher, the editors and the reviewers. Any product that may be evaluated in this article, or claim that may be made by its manufacturer, is not guaranteed or endorsed by the publisher.

## Abbreviations

CS: cardiogenic shock
DBP: diastolic blood pressure
CO: cardiac output
FA: femoral artery
IABP: intra-aortic balloon pump
MBP: mean blood pressure
MCS: mechanical circulatory support
MI: myocardial infarction
PAP: pulmonary artery pressure
PCI: percutaneous coronary intervention
PCWP: pulmonary capillary wedge pressure
SBP: systolic blood pressure
SVO_2_: mixed venous oxygen saturation
TcPO_2_: transcutaneous oxygen pressure.
