# Supplementary material for: Effectiveness and Safety of a Prolonged Hemodynamic Support by the IVAC2L System in Healthy and Cardiogenic Shock Pigs
Source: Front Cardiovasc Med. 2022 Feb 8;9:809143. doi: 10.3389/fcvm.2022.809143 (PMC8861279; doi:10.3389/fcvm.2022.809143)
Supplement: Supplementary file 1 [file Data_Sheet_1.docx]

Supplementary Material

# Supplementary Data

**Supplementary methods**

Ethics

Experiments were conducted in our certified laboratory (INSERM UMS-006 CREFRE Rangueil, agreement number C31 555 07).

All experiments were reviewed and approved by the national INSERM French Ethics Committee for animal experimentation (CE201609131807621V8). The procedure for the care and sacrifice of study animals was in accordance with the European Community Standards on the Care and Use of Laboratory Animals.

Anaesthetic protocol

Pigs were pre-medicated by intramuscular injection of ketamine (0.3 to 0.5mg.kg), azapirone (0.01-0.02 µg/kg) and midazolam (0.5-0.75 mg/kg). Anaesthesia was induced by propofol (0.5-1.5mg/kg) and myorelaxation was achieved by cisatracurium besilate (0.025-0.04 mg/kg) intravenous injection on a peripheral ear lobe vein.

Pigs were ventilated after oro-tracheal intubation (tube size 8.5 to 10 as needed) under assisted volume-controled mode (Evita XL Drager, Drager, Luebeck, Germany) with the following parameters: tidal volume 8 mL/kg, PEEP 5 cm H2O, initial FiO2 100% then adjusted to a saturation of 94–96%. Insuflation rate was adapted for PaCO2 between 35 and 45 mmHg or EtCO2 (NICO, Dixtal Biomedica Ind. Com, Sao Paulo, Brazil) between 30 and 40 mmHg.

Anaesthesia was maintained by midazolam (0.5-0.75 mg/kg/h), sufentanil (0.2-0.5 µg/kg/min) and propofol (2-4 mg/kg/h) infusion associated with continue myorelaxation by cisatracurium besilate (0.2-0.5 mg/kg/h).

An initial vascular filling by *NaCl 9%* isotonic 500mL was given after induction and continuous infusion was maintained (1-3 mL/kg/h). Additional filling tests and use of vasopressors (norepinephrine) were used as necessary to maintain mean blood pressure (MBP) between 65 and 80 mmHg

Antiarrhythmic preparation was made by amiodarone 300 mg over 30 minutes before the procedure and lidocaine 1mg/kg during experiments in order to prevent the risk of ventricular arrhythmias.

iVAC2L insertion and management

The right common femoral artery (FA) was exposed through a 10-cm oblique incision in the groin crease and was circumferentially controlled with a vessel loop. A purse string suture (5-0 Prolene) was placed on each of two puncture sites. Heparin was administered aiming for an activated clotting time over 250 seconds. Firstly, an anterograde puncture of the FA allowed the insertion of a 7 Fr Super Arrow-Flex^TM^ sheath (Teleflex, Wayne, PA). Secondly, a retrograde puncture was performed above the previous site; a 6 Fr arterial sheath was inserted then exchanged on a stiff 0.035” guidewire for an 19 Fr SoloPath^TM^ balloon expandable transfemoral system (Terumo Corp., Tokyo, Japan). Finally, Super Arrow-Flex^TM^ sheath was connected to the Solopath sheath, in order to perfuse the right inferior limb. The purse string sutures were knotted on the sheaths to safely secure it.

The subsequent steps were performed as previously described (13,17). A 6 Fr pigtail catheter was used for the retrograde crossing of the aortic valve and a pre-shaped 0.035” stiff guidewire (Amplatz Super Stiff^TM^ Guidewire; Boston Scientific, Marlborough, MA, USA) was positioned in the apex of the left ventricle under fluoroscopy and / or echocardiography. The bi-directional flow catheter was de-aired and advanced over the 0.035” stiff guidewire until the inlet tip has entered the left ventricle. Stiff guidewire and dilator were carefully removed, leaving the tip of the catheter in the LV apex. As the dilator was removed from the catheter, blood was bleeding back thanks to blood pressure and de-aired the system. The catheter was then temporarily squeezed with a surgical clamp. The chamber of the membrane pump was filled with heparinised saline and a wet-to-wet connection was made between the filled pump and the catheter. After confirmation of an air-free connection, the clamp was relieved. The extra-corporeal membrane pump was also connected to the IABP console (**Arrow ACAT 2 Wave**) which was then activated and synchronized to the animal thanks to EKG and invasive blood pressure monitoring, thus generating pulsatile flow.

In case of premature ventricular beats, intravenous injection of amiodarone (150-300 mg bolus) for each episode was made. In case of sustained ventricular disorders, injection of lidocaine (0.5-1mg/kg) was associated.

Groin incision was closed in anatomic layers and the catheter was firmly attached to the skin of the limb.

An initial intravenous bolus of UFH (30 UI/kg) was made at reperfusion insertion time and anticoagulation target (activated clotting time between 200 and 250s) was then maintained by intravenous infusion of UFH (2500 to 6000 UI/h).

## Monitoring

Non-invasive monitoring of heart rate, blood oxygen saturation, and EKG was continuously performed (Spacelabs® datascope). Invasive systolic (SBP), diastolic (DBP) and mean blood pressure were monitored by an arterial catheter in the left carotid (4Fr – 8mm). A Swan-Ganz catheter was echo-guided inserted on a 8Fr catheter in the right internal jugular vein to monitor pulmonary artery pressure (sPAP (systolic pulmonary artery pressure), mPAP (mean pulmonary artery pressure), dPAP (diastolic pulmonary artery pressure), PCWP (pulmonary capillary wedge pressure). CO (cardiac output) was measured using the thermodilution approach (Vigilance II, Edwards®) and obtained after injection of 10mL of cold isotonic saline 9% (<18°C) via the Swan-Ganz catheter.

Lower limb perfusion was clinically assessed (heat, cutaneous coloration time) and a continuously transcutaneous pressure in oxygen (TcpO2) monitoring was also used (TCM 400-2, Radiometer SAS®, France) during experiments (48 hours in healthy pigs).

Respiratory (arterial and venous blood gaz) and renal (creatinine, kaliemia, natremia, chloremia) functions, systemic and distal limb perfusion (lactate), hemostase (TQ, aPTT) were iteratively monitored by relocated biology monitor (EPOC, CoagPoc, EDGE®). Others biological sample were frozen to realized specific post hoc analysis (LDH and troponin).

# Supplementary Figures and Tables

## Supplementary Tables

**Supplementary table 1: Comparative analysis of invasive and non-invasive hemodynamic parameters between IVAC2L On vs Off in healthy pigs, presented as mean values of all the time points.**

| **Time** | **IVAC2L On** | | | **IVAC2L Off** | | | **p value** |
| --- | --- | --- | --- | --- | --- | --- | --- |
|  | **Mean** | **SEM** | **Number of times points** | **Mean** | **SEM** | **Number of times points** |  |
| **SBP (mmHg)** | 107.3 | 2.0 | 10 | 107.4 | 2.3 | 10 | 0.994 |
| **DBP (mmHg)** | 73.1 | 1.0 | 9 | 68.5 | 1.0 | 9 | **0.006** |
| **MBP (mmHg)** | 89.3 | 1.2 | 10 | 85.2 | 1.7 | 10 | 0.063 |
| **HR (bpm)** | 76 | 2 | 10 | 77 | 2 | 10 | 0.467 |
| **sPAP (mmHg)** | 23.7 | 0.6 | 10 | 24.1 | 0.7 | 10 | 0.685 |
| **dPAP (mmHg)** | 10.7 | 0.6 | 10 | 10.5 | 0.4 | 10 | 0.743 |
| **mPAP (mmHg)** | 16.4 | 0.6 | 10 | 16.4 | 0.5 | 10 | > 0.999 |
| **PCWP (mmHg)** | 11.6 | 0.3 | 10 | 12.8 | 0.3 | 10 | **0.018** |
| **CO (l/min)** | 5.39 | 0.28 | 9 | 5.2 | 0.3 | 9 | 0.624 |

Unpaired t-test was used to compare each data IVAC2L On vs Off at each point.

CO, cardiac output; DBP, diastolic blood pressure; dPAP, diastolic pulmonary artery pressure; HR, heart rate; MBP, mean blood pressure; mPAP, mean pulmonary artery pressure; SBP, systolic blood pressure; SEM, standard error of mean; PCWP, pulmonary capillary wedge pressure; sPAP, systolic pulmonary artery pressure

**Supplementary table 2: Comparative analysis of right and left inferior legs transcutaneous oxygen pressure before and after iVAC2Limplantation in healthy pigs**

|  | **RIL TcPO2** | | | **LIL TcPO2** | | | **p value** |
| --- | --- | --- | --- | --- | --- | --- | --- |
|  | Mean | SEM | n | Mean | SEM | n |  |
| **T0** | 225.7 | 64.1 | 6 | 191.7 | 63.9 | 6 | 0.72 |
| **Off + 10min** | 26.7 | 8.6 | 6 | 165.7 | 53.6 | 6 | **0.03** |
| **Off + 30min** | 4.8 | 2.8 | 6 | 128.7 | 42.4 | 6 | **0.02** |
| **Off + H1** | 2.3 | 0.6 | 6 | 116.7 | 29.9 | 6 | **0.00** |
| **H1 On** | 14.3 | 5.6 | 6 | 95.7 | 24.1 | 6 | **0.01** |
| **H2 On** | 17.8 | 5.0 | 6 | 89.3 | 23.7 | 6 | **0.01** |
| **H4 On** | 19.6 | 10.9 | 5 | 79.4 | 18.6 | 5 | **0.02** |
| **H8 On** | 42.0 | 20.3 | 5 | 82.2 | 8.3 | 5 | 0.10 |
| **H12 On** | 37.0 | 12.9 | 5 | 74.0 | 3.7 | 4 | **0.04** |
| **H24 On** | 56.5 | 27.7 | 4 | 116.5 | 14.0 | 4 | 0.10 |
| **H36 On** | 34.8 | 7.7 | 4 | 71.0 | 9.1 | 3 | **0.03** |
| **H 48 On** | 73.3 | 36.9 | 3 | 91.0 | 26.5 | 3 | 0.72 |

All TcPO2 values were in mmHg. Paired t-test was used to compare RIL and LIL TcPO2 at each point.

T0, basal measure after sedation; H2 On, 2 hour post iVAC2L start; H4 On, 4 hours post iVAC2L start; H8 On, 8 hours post iVAC2L start; H12 On, 12 hours post iVAC2L start; H24 On, 24 hours post iVAC2L start; H36 On, 36 post iVAC2L start; H48 On, 48 post iVAC2L start; Off, Solopath inserted but reperfusion Off; LIL, left inferior leg; n, number of pigs; RIL, right inferior leg; TcPO2, transcutaneous oxygen pressure

**Supplementary table 3: Comparative analysis of systemic, right and left inferior legs lactate evolution before and after iVAC2L implantation in healthy pigs**

| **Time** | **RIL lactate (mmol/l)** | | | **LIL lactate (mmol/l)** | | | **Systemic lactate (mmol/l)** | | | **p value (RIL vs LIL)** | **p value (RIL vs Systemic)** | **p value (LIL vs Systemic)** |
| --- | --- | --- | --- | --- | --- | --- | --- | --- | --- | --- | --- | --- |
|  | Mean | SEM | n | Mean | SEM | n | Mean | SEM | n | **-** | **-** | **-** |
| **T0** | - | - | - | - | - | - | 0.88 | 0.36 | 6 | **-** | **-** | **-** |
| **Off + 10min** | 6.64 | 1.13 | 5 | - | - | - | 1.78 | 0.49 | 5 | **-** | **<0.01** |  |
| **Off + H1** | 8.37 | 0.82 | 6 | 1.93 | 0.33 | 6 | 1.68 | 0.32 | 6 | **< 0.0001** | **< 0.01** | 0.59 |
|  | | | | | | | | | | | | |
| **H2 On** | 4.73 | 1.07 | 6 | 2.33 | 0.68 | 5 | 1.61 | 0.47 | 5 | **0.10** | **0.03** | 0.41 |
| **H4 On** | 3.17 | 0.48 | 3 | 2.40 | 1.10 | 2 | 0.60 | 0.35 | 3 | **0.51** | **0.01** | 0.15 |
| **H8 On** | 1.55 | 0.23 | 3 | 0.78 | 0.23 | 2 | 0.23 | 0.23 | 3 | **0.10** | **0.02** | 0.21 |
| **H12 On** | 1.68 | 0.15 | 4 | 1.03 | 0.23 | 3 | 0.48 | 0.10 | 4 | **0.06** | **< 0.01** | **0.06** |
| **H24 On** | 1.43 | 0.87 | 2 | 1.67 | 0.19 | 3 | 0.40 | 0.05 | 4 | **0.75** | 0.13 | **< 0.01** |
| **H36 On** | 3.08 | 0.38 | 4 | 1.10 | 0.56 | 3 | 0.19 | 0.11 | 4 | **0.03** | **< 0.01** | 0.12 |
| **H 48 On** | 3.40 | 1.11 | 3 | 1.87 | 0.57 | 3 | 0.39 | 0.09 | 3 | **0.29** | **0.05** | 0.06 |

Paired t-test was used to compare RIL and LIL, RIL to systemic and LIL to systemic lactate respectively at each point.

T0, basal measure after sedation; H2 On, 2 hour post iVAC2Lstart; H4 On, 4 hours post iVAC2L start; H8 On, 8 hours post iVAC2L start; H1 On2, 12 hours post iVAC2L start; H24 On, 24 hours post iVAC2L start; H36 On, 36 hours post iVAC2L start; H48 On, 48 hours post iVAC2L start; Off, Solopath inserted but reperfusion Off; LIL, left inferior leg; n, number of pigs; RIL, right inferior leg

**Supplemental table 4: LDH evolution under iVAC2L support in healthy pigs**

| **Time** | **LDH (mIU/mL)** | | | **p-value (vs H0)** |
| --- | --- | --- | --- | --- |
|  | Mean | SEM | n |  |
| **H0** | 6.357 | 1.032 | 5 | **-** |
| **H12** | 6.920 | 1.318 | 3 | 0.749 |
| **H24** | 11.629 | 1.265 | 4 | **0.014** |
| **H36** | 13.443 | 4.097 | 3 | 0.075 |
| **H48** | 13.893 | 2.954 | 3 | **0.018** |

Paired t-test was used to compare each point to H0.

T0, baseline measure after sedation; H12 On, 12 hours post iVAC2L start; H24 On, 24 hours post iVAC2L start; H36 On, 36 post iVAC2L start; H48 On, 48 post iVAC2L start

**Supplementary table 5: Comparative analysis of systemic, right inferior leg and left inferior leg lactate before and during cardiogenic shock under iVAC2L support**

| **Time** | **Systemic lactate (mmol/l)** | | | **LIL lactate (mmol/l)** | | | **RIL lactate (mmol/l)** | | | **p-value**  (RIL vs LIL) | **p-value** (Systemic vs RIL) | **p-value** (Systemic vs LIL) |
| --- | --- | --- | --- | --- | --- | --- | --- | --- | --- | --- | --- | --- |
|  | Mean | SEM | n | Mean | SEM | n | Mean | SEM | n |  |  |  |
| **T0** | 1.58 | 0.27 | 4 | 2.05 | 0.38 | 4 | 2.03 | 0.25 | 4 | 0.958 | 0.271 | 0.353 |
| **Shock** | 3.90 | 0.46 | 4 | - | - | - | - | - | - | - | - | - |
| **H1** | 0.83 | 0.28 | 4 | 2.05 | 0.44 | 4 | 2.58 | 0.65 | 4 | 0.529 | **0.048** | 0.057 |
| **H4** | 2.00 | 0.41 | 4 | 1.60 | 0.21 | 4 | 2.18 | 0.61 | 4 | 0.408 | 0.820 | 0.418 |
| **H6** | 0.28 | 0.28 | 4 |  |  |  |  |  |  |  |  |  |
| **H8** | 0.58 | 0.33 | 4 |  |  |  |  |  |  |  |  |  |
| **H10** | 1.15 | 1.15 | 4 |  |  |  |  |  |  |  |  |  |
| **H12** | 0.83 | 0.83 | 4 | 1.65 | 0.79 | 4 | 2.55 | 0.65 | 4 | 0.413 | 0.152 | 0.497 |

Paired t-test was used to compare RIL and LIL, RIL to systemic and LIL to systemic lactate respectively at each point.

T0, basal measure after sedation; H1, 1 hour post cardiogenic shock; H4, 4 hours after cardiogenic shock; H6, 6 hours post cardiogenic shock; H8, 8 hours after cardiogenic shock; H10, 10 hours post cardiogenic shock; H12, 12 hours after cardiogenic shock; LIL, left inferior leg; n, number of pigs; RIL, right inferior leg; SEM, standard error of the mean

## Figures

**Supplementary figure 1: Comparative analysis of systolic blood pressure (Figure 1A), mean blood pressure (Figure 1B) and diastolic blood pressure (Figure 1C) evolution during 48h of support with iVAC2L support (On vs Off) in healthy pigs**

**

**

At different times (H0, H2, H4, H6, H8, H10, H12, H16, H24, H36 and H48), the IVAC2L support was deactivated by turning Off the extra-corporal pump during 5 minutes and then reactivated until next time point. Invasive and non-invasive hemodynamic parameters were recorded throughout the procedure and until 48 hours. Parameters are presented at each time points by mean and SEM.

CO, cardiac output; HR, heart rate; PCWP, pulmonary capillary wedge pressure; SEM, standard error of mean

Parameters are presented as mean and SEM.

DBP, diastolic blood pressure; MBP, mean blood pressure; SBP, systolic blood pressure; SEM, standard of mean

**Supplemental figure 2: Comparative analysis of systolic pulmonary artery pressure (Figure 2A), mean pulmonary artery pressure (Figure 2B) and diastolic pulmonary artery pressure (Figure 2C) evolution during 48h of support with iVAC2L support (On vs Off) in healthy pigs**

**

**

At different times (H0, H2, H4, H6, H8, H10, H12, H16, H24, H36 and H48), the IVAC2L support was deactivated by turning Off the extra-corporal pump during 5 minutes and then reactivated until next time point. Invasive and non-invasive hemodynamic parameters were recorded throughout the procedure and until 48 hours. Parameters are presented at each time points by mean and SEM.

CO, cardiac output; HR, heart rate; PCWP, pulmonary capillary wedge pressure; SEM, standard error of mean

Parameters are presented as mean and SEM.

dPAP, diastolic pulmonary artery pressure; mPAP, mean pulmonary artery pressure; SEM, standard of mean; sPAP, systolic pulmonary artery pressure

**Supplementary figure 3: LDH evolution under iVAC2L support in healthy pigs**

**

**

Parameters are presented as mean and SEM. Paired t-test was used to compare each point to H0.

* p < 0.05 vs T0

T0, baseline measure after sedation; H12 On, 12 hours post iVAC2L start; H24 On, 24 hours post iVAC2L start; H36 On, 36 post iVAC2L start; H48 On, 48 post iVAC2L start; SEM, standard error of the mean

**Supplementary figure 4: Troponin evolution after left anterior descending coronary artery, shock and under iVAC2L support in cardiogenic shock pigs**





Parameters are presented as mean and SEM. Paired t-test was used to compare each point to H0.

* p < 0.05 vs H0

H0, baseline measure; H4, 4 hours after shock and iVAC2L start; H12, 12 hours after shock and iVAC2L start; LAD, left anterior descending coronary

**Supplementary figure 5: Right inferior leg lactate evolution during support in cardiogenic shock pigs**

**

**

Parameters are presented as mean. Paired t-test was used to compare each point to H0.

All comparison with H0 were non-significant

H0, baseline measure; H1, 1 hours after shock and iVAC2L start; H4, 4 hours after shock and iVAC2L start; H12, 12 hours after shock and iVAC2L start; RIL, right inferior leg

**Supplementary figure 6: LDH evolution under iVAC2L support in cardiogenic shock pigs**

**

**

Parameters are presented as mean and SEM. Paired t-test was used to compare each point to H0.

* p < 0.05 vs H0

H0, baseline measure; H1, 1 hours after shock and iVAC2L start; H4, 4 hours after shock and iVAC2L start; H12, 12 hours after shock and iVAC2L start; SEM, standard error of the mean

**Supplementary figure 7: Hemoglobin evolution during iVAC2L support in cardiogenic shock pigs**

**

**

Parameters are presented as mean and SEM. Paired t-test was used to compare each point to H0.

* p < 0.05 vs H0

H0, baseline measure; H1, 1 hours after shock and iVAC2L start; H4, 4 hours after shock and iVAC2L start; H12, 12 hours after shock and iVAC2L start
